# The Housing Campaign

**Published:** 1920-05-08

**Authors:** 


### The Housing Campaign.

### Great National Effort Required.

The urgency and at the same time the difficulty of the housing problem were clearly demonstrated at the Guildhall meeting addressed by Mr. Bonar Law in support of local authorities' housing bonds. There was a shortage of houses before the war, and operations were suspended during the war. Now, when all that leeway has to be made up, there is a shortage of raw material, a shortage of labour, and money stringency. Yet the provision of housing is a vital, social, and indeed, political problem. It affects all classes, and it falls with particular hardness upon ex-Service men. It gives rise to discontent and bitterness, and the "interests of public health and humanity are at stake." A great effort must seriously be made. The figures given by Mr. Bonar Law of the latest progress are not too encouraging—18,000 local housing proposals approved, tenders approved for 100,000, and the number of houses actually in course of erection 30,000. But the need must be met, and the duty laid upon patriotism is as great now as it was during the war. Money is wanted for everything, both locally and nationally, but public spirit ought not to be lacking in the provision of what is urgent. Housing is certainly high up in that category. The Minister of Health says there are £2,000,000,000 on deposit in the banks, and what is wanted for housing during the next twelve months is not one-tenth of that sum. Some of it might be spared for a trustee investment at six per cent. It is perhaps not easy to arouse enthusiasm for local investments when profitable national investments are available, and it may prove to be that local bonds are not altogether wise for that reason. But the scheme is on foot, and it is a patriotic obligation to support with all possible energy the sincere efforts of the Ministry of Health to grapple with the situation. It will be worthless to abuse the authorities if the community does not play its own individual part, and with one or two querulous exceptions there has been shown a whole-hearted desire to give the Ministry a fair field, to forget past mistakes, and to stimulate effort by useful and constructive criticism. It is fatuous to crab the housing bond scheme, as the *Daily Herald* does, because the minority of extremists which that paper represents are dissatisfied with the present order of civilisation. It is folly to sit still doing nothing but to cry for the moon, especially when those who cry are the ones who suffer as much as any.

